# Analysis of left ventricle regional myocardial motion for cardiac radioablation: Left ventricular motion analysis

**DOI:** 10.1002/acm2.14333

**Published:** 2024-03-17

**Authors:** Justin Poon, Richard B. Thompson, Marc W. Deyell, Devin Schellenberg, Haley Clark, Stefan Reinsberg, Steven Thomas

**Affiliations:** ^1^ Department of Physics and Astronomy University of British Columbia Vancouver British Columbia Canada; ^2^ Department of Medical Physics BC Cancer Vancouver British Columbia Canada; ^3^ Department of Biomedical Engineering University of Alberta Edmonton Alberta Canada; ^4^ Heart Rhythm Services Division of Cardiology University of British Columbia Vancouver British Columbia Canada; ^5^ Department of Radiation Oncology BC Cancer Surrey British Columbia Canada; ^6^ Department of Medical Physics BC Cancer Surrey British Columbia Canada

**Keywords:** cardiac motion, cardiac radioablation, cardiac radiosurgery, cardiac SBRT, STAR

## Abstract

**Purpose:**

Left ventricle (LV) regional myocardial displacement due to cardiac motion was assessed using cardiovascular magnetic resonance (CMR) cine images to establish region‐specific margins for cardiac radioablation treatments.

**Methods:**

CMR breath‐hold cine images and LV myocardial tissue contour points were analyzed for 200 subjects, including controls (*n* = 50) and heart failure (HF) patients with preserved ejection fraction (HFpEF, *n* = 50), mid‐range ejection fraction (HFmrEF, *n* = 50), and reduced ejection fraction (HFrEF, *n* = 50). Contour points were divided into segments according to the 17‐segment model. For each patient, contour point displacements were determined for the long‐axis (all 17 segments) and short‐axis (segments 1–12) directions. Mean overall, tangential (longitudinal or circumferential), and normal (radial) displacements were calculated for the 17 segments and for each segment level.

**Results:**

The greatest overall motion was observed in the control group—long axis: 4.5 ± 1.2 mm (segment 13 [apical anterior] epicardium) to 13.8 ± 3.0 mm (segment 6 [basal anterolateral] endocardium), short axis: 4.3 ± 0.8 mm (segment 9 [mid inferoseptal] epicardium) to 11.5 ± 2.3 mm (segment 1 [basal anterior] endocardium). HF patients exhibited lesser motion, with the smallest overall displacements observed in the HFrEF group—long axis: 4.3 ± 1.7 mm (segment 13 [apical anterior] epicardium) to 10.6 ± 3.4 mm (segment 6 [basal anterolateral] endocardium), short axis: 3.9 ± 1.3 mm (segment 8 [mid anteroseptal] epicardium) to 7.4 ± 2.8 mm (segment 1 [basal anterior] endocardium).

**Conclusions:**

This analysis provides an estimate of epicardial and endocardial displacement for the 17 segments of the LV for patients with normal and impaired LV function. This reference data can be used to establish treatment planning margin guidelines for cardiac radioablation. Smaller margins may be used for patients with higher degree of impaired heart function, depending on the LV segment.

## 1 INTRODUCTION

Ventricular tachycardia (VT) is a rapid heart rhythm that can be life threatening when occurring in the setting of diseased myocardial tissue. Scarred myocardium resulting from previous injury, such as myocardial infarction, can create re‐entrant electrical circuits that cause VT, and this arrhythmia can lead to reduced cardiac output or sudden cardiac death.^1^, ^2^ Implantable cardioverter‐defibrillator (ICD) therapy can be used to deliver an electric shock that resets the heart rhythm and prevents sudden cardiac arrest. However, this is painful and does not prevent future VT episodes. In cases of recurrent VT, antiarrhythmic drug therapy is first line therapy but is not always effective and may result in side effects or serious toxicity.^3^, ^4^ Catheter ablation may be more effective, but its invasive nature increases risk of procedural complications and it has limitations, particularly for targeting deeper myocardial scar and more widespread scar.^5^, ^6^, ^7^, ^8^

Cardiac radioablation is a promising non‐invasive treatment modality for VT, where a medical linear accelerator is used to irradiate the arrhythmogenic region within the heart.^9^, ^10^, ^11^, ^12^, ^13^, ^14^, ^15^ However, when delivering radiation dose to a region within the heart, the treatment margin must be sufficiently large to account for respiratory and cardiac motion. The re‐entrant transmitting volume (RTV) is the targeted scar region where re‐entrant electrical circuits occur, otherwise referred to as the clinical target volume (CTV). An internal margin is added to encompass the targeted tissue and its full range of motion, creating the internal target volume (ITV). The ITV is most often established using respiratory‐binned 4DCT to quantify the target motion.^15^, ^16^ The ITV is created from a composite of target volumes delineated on each respiratory phase of the 4DCT, or may be generated from a maximum or average intensity projection (maximum or average pixel densities for all phase images). An additional 2 to 5 mm is then added to create the planning target volume (PTV), which accounts for set‐up uncertainty and treatment delivery inaccuracies. However, the ability to properly capture the cardiac‐induced motion in respiratory 4DCT is debatable, as blurring in the 4DCT images due to cardiac contraction might lead to inaccurate motion assessment and target volume delineation variability. Region‐specific cardiac‐induced motion data would be valuable for improved treatment margin definition, especially for targets that have large cardiac‐induced motion or are proximal to critical organs at risk (OARs). The motion of the myocardium over the cardiac cycle is also complex and heterogeneous, so creating an ITV with treatment margins appropriate to the targeted heart region is expected to improve dose conformity and minimize the dose delivered to surrounding healthy tissue. Additionally, in patients with globally or regionally impaired function, the range of motion may be reduced which provides an opportunity for smaller ITVs.

Previous regional cardiac motion studies have mostly focused on measurements in healthy hearts.^17^, ^18^, ^19^, ^20^, ^21^, ^22^ Studies involving patients with impaired hearts have focused on myocardial strain measurements.^23^, ^24^ In a recent review by Stevens et al., average cardiorespiratory motion of target volumes for cardiac radioablation was summarized.^16^ The review suggests that despite increased numbers of VT patients being treated, detailed cardiorespiratory motion data is still limited. Large variations in motion between patients are noted, possibly explained by the fact that most VT patients suffer from heart failure (HF) and have varying levels of cardiac function. The authors also suggest that comparing the motion of targets in different regions of the heart would be valuable for investigating the feasibility of regional treatment margins.

The aim of this study was to assess left ventricle (LV) regional myocardial displacement due to cardiac motion using cardiovascular magnetic resonance (CMR) cine images across a wide range of LV function, including different HF groups with varying levels of left ventricular ejection fraction (LVEF). Epicardial (outer border) and endocardial (inner border) displacements are reported in the overall, longitudinal, circumferential, and radial directions for each segment of the LV.^25^ This will provide a LV function‐dependent and region‐specific reference dataset to aid in cardiac radioablation treatment planning. While patient‐specific motion data is always preferable, especially since cardiac motion can have large inter‐patient variability, the data from this study serves as a valuable reference for typical cardiac‐induced motions that are expected to be seen in each LV segment. Many treatments have used respiratory‐binned 4DCT to evaluate the target motion, where cardiac‐induced motion is only represented as a blur in the images. However, this blur may not accurately capture the entire cardiac‐induced motion—for example, motion tangential to the myocardial borders (longitudinal and circumferential) cannot be visualized. In cases where patient‐specific cardiac‐induced motion data is not available, this reference data may provide an additional tool for guiding margin decisions.

## 2 METHODS

CMR images and LV myocardial tissue tracked contour points for 200 individuals were analyzed.^24^ Epicardial (outer border) and endocardial (inner border) displacements in all directions were characterized for the regions of the LV defined by the 17‐segment model^25^ and for four subject subgroups: a combined control group (25 healthy controls and 25 at‐risk for HF and HF patients with preserved ejection fraction (HFpEF > 55%, *n* = 50), mid‐range ejection fraction (HFmrEF 40% to 55%, *n* = 50), and reduced ejection fraction (HFrEF < 40%, *n* = 50), with similar numbers of male and female subjects (Table 1). The at‐risk group consisted of patients with one or more of the following: hypertension, diabetes, obesity, atrial fibrillation, chronic coronary artery disease, or chronic obstructive pulmonary disease. Subjects were chosen from the Alberta Heart Failure Etiology and Analysis Research Team (HEART) study^26^ (approved by the Health Research Ethics Boards at the University of Alberta and University of Calgary, ClinicalTrials.gov NCT02052804) by selecting the first 25 males and first 25 females for each of the control, HFpEF, and HFmrEF groups. For the HFrEF group, the 18 available females and the first 32 males were selected. Subjects were originally recruited between 2010 and 2014. The subgroups represent a wide range of structural abnormalities, such as ventricular hypertrophy or dilation, and a wide range of function with both global and regional abnormalities. VT patients eligible for cardiac radioablation generally have lower LVEF levels.^13^, ^27^

**TABLE 1:** Subject group demographics.

	Controls (*n* = 50)	HFpEF (*n* = 50)	HFmrEF (*n* = 50)	HFrEF (*n* = 50)	*p*‐Value
**Age**	62.0 ± 9.5	71.7 ± 9.7^a^	64.6 ± 13.8^b^	66.3 ± 11.5	<0.001
**Male**	25 (50%)	25 (50%)	25 (50%)	32 (64%)	0.399
**Height (cm)**	170.0 ± 10.7	167.5 ± 10.4	168.5 ± 9.4	172.1 ± 10.2	0.133
**Weight (kg)**	79.3 ± 15.8	87.9 ± 18.6	82.5 ± 17.9	86.6 ± 20.8	0.081
**BMI (kg/m^2^)**	27.4 ± 5.2	31.2 ± 5.8^a^	28.9 ± 5.4	29.0 ± 5.5	0.008
**BSA (m^2^)**	1.9 ± 0.2	2.0 ± 0.3	2.0 ± 0.2	2.0 ± 0.3	0.194
**NYHA Class**		2.1 ± 0.7	2.0 ± 0.7	2.0 ± 0.7	0.756
**I**		10 (20%)	12 (24%)	10 (20%)	
**II**		27 (54%)	23 (46%)	28 (56%)	
**III**		13 (26%)	10 (20%)	11 (22%)	
**N/A**			5 (10%)	1 (2%)	
**LVEF (%)**	61.9 ± 5.4	63.0 ± 4.8	47.4 ± 4.1^a^, ^b^	30.6 ± 6.9^a^, ^b^, ^c^	<0.001

*Note*: Values are mean ± standard deviation or N (%). *p*‐Values were derived from chi‐square for gender or ANOVA for the continuous variables, and post‐hoc comparisons between groups were carried out using the Holm‐Bonferroni method.

Abbreviations: BMI, body mass index; BSA, body surface area; NYHA, New York Heart Association; N/A, not available (NYHA class not indicated).

^a^ Significantly different from controls.

^b^ Significantly different from HFpEF.

^c^ Significantly different from HFmrEF.

### 2.1 CMR data

Images were acquired on 1.5 T CMR scanners (Siemens Sonata or Avanto, Siemens Healthcare, Erlangen, Germany) using standard balanced steady state free precession (bSSFP) cine imaging with the following acquisition parameters: repetition time/echo time (TR/TE) 2.8/1.4 ms, 50−70‐degree flip angle, 8 mm slice thickness, 25 or 30 reconstructed cardiac phases. Images were acquired with electrocardiographic gating within an 8−12 s breath‐hold per slice. Each patient scan included two short‐axis slices (basal and/or mid‐cavity) and two‐, three‐, and four‐chamber view long‐axis slices.

Epicardial and endocardial borders of the LV were first manually traced on the end‐diastolic image frame. Border points were then propagated to all image frames over the cardiac cycle using feature tracking displacement fields (calculated from non‐rigid registration of all image frames to the reference end‐diastolic image frame).^28^ For each cardiac phase, equally spaced points along the borders were generated from the original points using spline interpolation (500 and 120 points per border for long‐axis and short‐axis slices, respectively). Further details on CMR data and image analysis are provided by Xu et al.^24^

### 2.2 Contour point motion analysis

**FIGURE 1:**
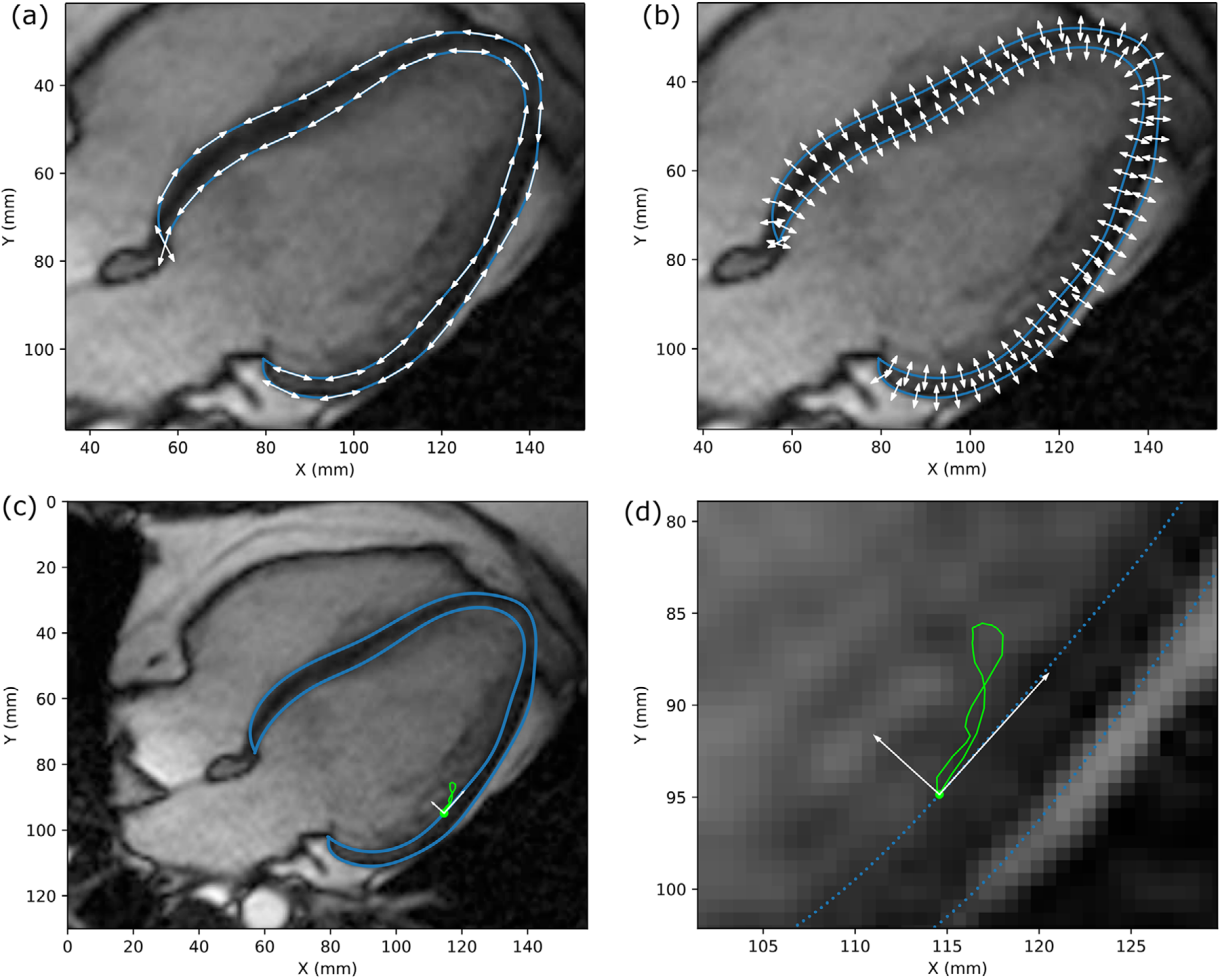
Illustration of unit vectors used to determine epicardial and endocardial displacement tangential/longitudinal (a) and normal/radial (b) to the border. An example of the tangential and normal components of a contour point's displacement is shown in (c). The close‐up view of the contour point and its motion trace is shown in (d).

Motion analysis was performed one slice at a time using Python code written in‐house (external libraries used: numpy,^29^ scipy,^30^ pandas,^31^, ^32^ statsmodels^33^). Contour point data in pixel coordinates was converted to millimeter (mm) coordinates using the slice's pixel spacing information. Contour point data was also converted to three‐dimensional (3D) coordinates using the slice's LPS (left‐posterior‐superior) data, which defines the image coordinates in 3D space relative to the patient coordinate system (axial, coronal, and sagittal planes). The maximum displacement for each contour point was determined by calculating the maximum distance between corresponding contour point positions from any two cardiac phases. Similarly, the maximum contour point displacements in the directions tangential (longitudinal for long‐axis, circumferential for short‐axis) and normal (radial) to the epicardium and endocardium contours were determined by taking scalar projections onto the unit tangent and normal vectors at each point location (Figure 1). Unit vectors were calculated from the derivatives along the spline‐interpolated curves. Visual examples of the contour point displacements are also shown in Video S1.

### 2.3 Contour point segmentation

**FIGURE 2:**
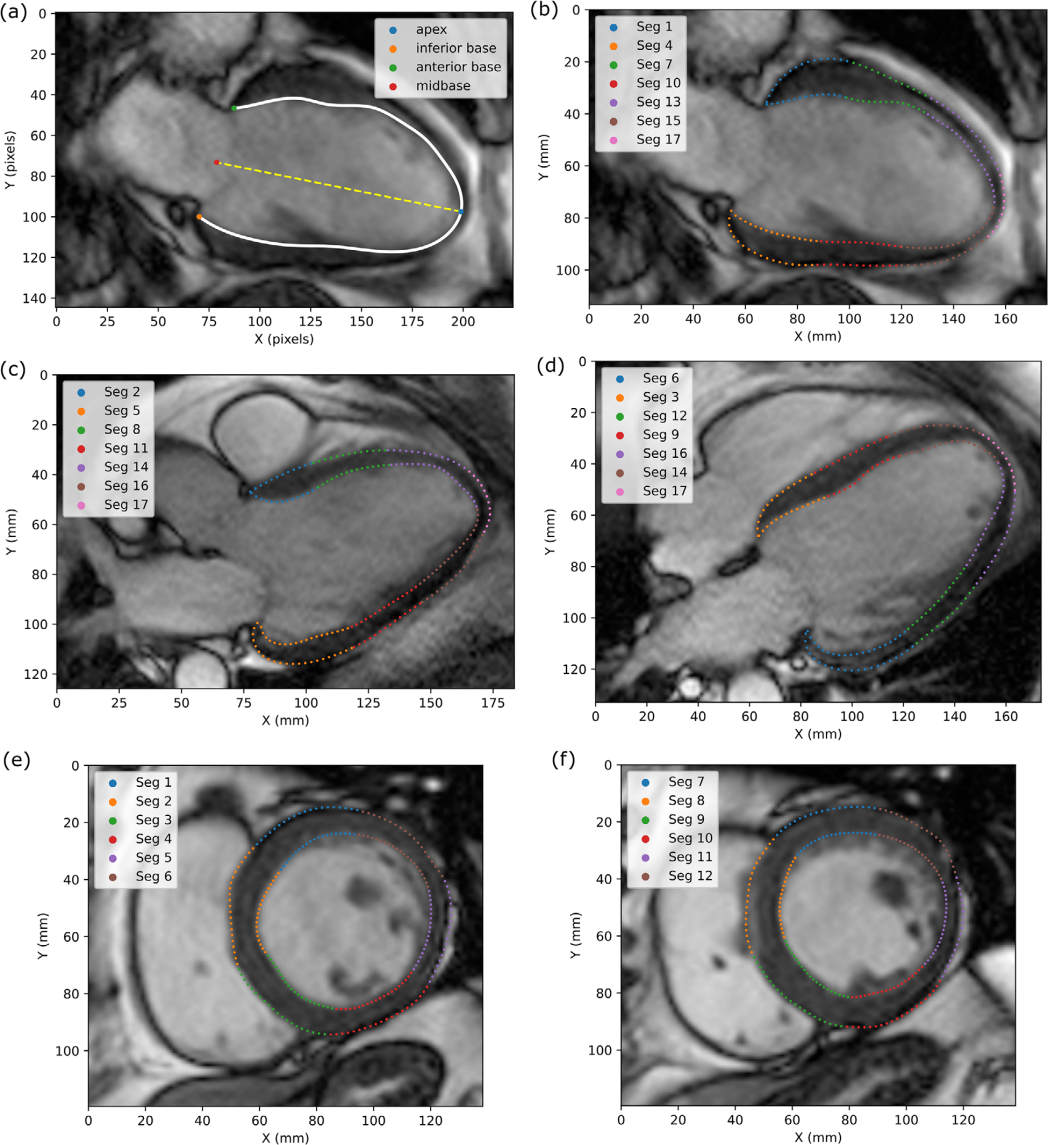
Contour point segmentation for a sample patient. (a) Shows an example of the automatically defined LV long axis used for the segmentation. (b), (c), and (d) show the resulting segmentation of the two‐chamber, three‐chamber, and four‐chamber view long‐axis slices, respectively. (e) and (f) show the segmentation for basal and mid‐cavity short‐axis slices, respectively. Only a subset of the contour points is shown for visibility purposes.

Contour points were automatically segmented based on the slice view and the long axis of the LV (Figure 2a). Figure 2b–d show the segmented points for the long‐axis slices, and Figure 2e–f show the short‐axis slices. Visual examples of the segmented points are also shown in Video S2. A detailed description of the segmentation process is included in the Supplementary Materials.

From the segmented contour points, mean epicardial and endocardial displacements for each segment were calculated. The overall displacement (within the slice plane), tangential displacement (longitudinal for long‐axis, circumferential for short‐axis), and normal displacement (radial direction) were separately determined.

### 2.4 Patient analysis

Baseline characteristics for the patient groups were compared using chi‐square or ANOVA where appropriate, and post‐hoc significance tests were performed using the Holm‐Bonferroni method. After acquiring displacement data for all 200 patients, the mean segmental displacements were determined for the four groups: controls (combined healthy controls and at‐risk for HF), HFpEF, HFmrEF, and HFrEF (50 subjects per group). For each segment, ANOVA and Holm‐Bonferroni post‐hoc analysis were used to compare the mean epicardial/endocardial displacements between groups. A *p*‐value less than 0.05 was considered to be statistically significant. This comparison provides additional context to the reference dataset by highlighting segments where motion is significantly affected in HF patients.

**FIGURE 3:**
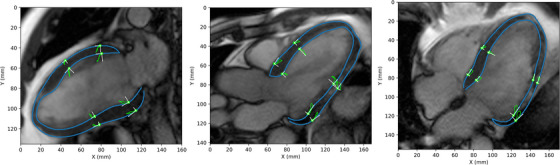
Contour point motion traces and the radial and longitudinal components were plotted for epicardial and endocardial basal and mid‐cavity segments. Motions originate from end diastole and are shown for all long‐axis slices of one HFrEF patient.

The general direction of epicardial and endocardial motion from the reference end‐diastolic phase was also investigated by looking at the motion traces of long‐axis slice contour points for four male and four female HFrEF patients. Motion traces and the corresponding radial and longitudinal components were plotted for the midpoint of each basal and mid‐cavity epicardial and endocardial segment (Figure 3). Motion trace figures for all eight patients are included in the Supplementary Material.

## 3 RESULTS

Table 1 outlines the demographic data for each of the subject groups. For each HF patient group, the majority were NYHA class II (slight limitation of physical activity) and none were categorized as class IV (symptoms at rest, discomfort with any physical activity). There was no significant difference between the groups for height, weight, body surface area (BSA), or mean NYHA class. For body mass index (BMI), only the HFpEF group was significantly larger than the controls. BSA was calculated using the Mosteller^34^ formula: BSA (m^2^) = (height (cm) x weight (kg)/3600)^½^.

Long‐axis epicardial and endocardial displacement data (overall/longitudinal/radial) are shown for the 17 segments and for each patient group in Tables 2, 3, and 4. Results are presented as mean ± standard deviation. The apical cap (segment 17) does not have endocardial points. Displacements are generally largest in the basal segments and decrease towards the apex. Significant motion reduction for the HF groups compared to the control group is also seen more often in the basal segments than towards the apical end.

**TABLE 2:** Long axis overall displacement (mean ± standard deviation) for each LV segment.

	Epicardium					Endocardium				
Segment	Control	HFpEF (>55%)	HFmrEF (40−55%)	HFrEF (<40%)	*p*‐Value	Control	HFpEF (>55%)	HFmrEF (40−55%)	HFrEF (<40%)	*p*‐Value
1 – basal anterior	11.3 ± 2.5	8.3 ± 3.0^a^	8.6 ± 2.9^a^	8.2 ± 2.9^a^	<0.001	12.2 ± 3.0	9.4 ± 3.1^a^	9.1 ± 3.2^a^	8.5 ± 3.1^a^	<0.001
2 – basal anteroseptal	7.9 ± 2.4	6.6 ± 2.0^a^	6.2 ± 2.0^a^	6.0 ± 2.0^a^	<0.001	6.9 ± 1.9	5.9 ± 1.6^a^	5.7 ± 1.6^a^	5.7 ± 1.7^a^	0.001
3 – basal inferoseptal	8.6 ± 2.5	6.7 ± 2.3^a^	6.2 ± 2.3^a^	5.8 ± 2.3^a^	<0.001	8.3 ± 2.3	6.5 ± 2.1^a^	6.2 ± 2.1^a^	5.9 ± 2.4^a^	<0.001
4 – basal inferior	12.6 ± 3.4	10.2 ± 3.6^a^	10.3 ± 3.4^a^	8.3 ± 2.8^a^, ^b^, ^c^	<0.001	12.3 ± 2.9	10.3 ± 3.4^a^	10.0 ± 3.3^a^	8.6 ± 2.7^a^, ^b^, ^c^	<0.001
5 – basal inferolateral	12.8 ± 2.5	12.1 ± 3.3	11.3 ± 3.4	9.9 ± 2.9^a^, ^b^	<0.001	13.2 ± 2.7	12.3 ± 3.1	11.1 ± 3.6^a^	10.0 ± 3.0^a^, ^b^	<0.001
6 – basal anterolateral	13.4 ± 3.1	12.0 ± 3.4	11.6 ± 3.6	10.4 ± 3.3^a^	<0.001	13.8 ± 3.0	12.7 ± 3.3	12.1 ± 3.4^a^	10.6 ± 3.4^a^, ^b^	<0.001
7 – mid anterior	6.9 ± 1.7	5.6 ± 2.1^a^	5.4 ± 2.1^a^	5.8 ± 2.3^a^	0.002	9.5 ± 2.4	8.8 ± 2.7	7.2 ± 2.6^a^,^b^	6.3 ± 2.7^a^, ^b^	<0.001
8 – mid anteroseptal	5.2 ± 1.5	4.9 ± 1.3	4.5 ± 1.7	4.8 ± 1.6	0.144	7.8 ± 1.8	7.4 ± 2.0	6.0 ± 2.0^a^, ^b^	5.2 ± 2.0^a^, ^b^	<0.001
9 – mid inferoseptal	5.8 ± 1.7	5.1 ± 1.8	4.7 ± 1.7^a^	4.7 ± 1.8^a^	0.005	7.7 ± 1.7	7.1 ± 2.1	5.8 ± 1.7^a^, ^b^	5.2 ± 2.0^a^, ^b^	<0.001
10 – mid inferior	9.3 ± 2.5	8.0 ± 2.9	8.1 ± 2.7	6.9 ± 2.2^a^	<0.001	10.1 ± 1.8	9.2 ± 2.5	8.3 ± 2.4^a^	7.0 ± 2.3^a^, ^b^, ^c^	<0.001
11 – mid inferolateral	9.6 ± 1.8	10.0 ± 2.9	9.4 ± 2.9	8.5 ± 3.0	0.066	10.1 ± 2.0	10.4 ± 2.7	9.3 ± 3.0	8.8 ± 3.3	0.018
12 – mid anterolateral	10.0 ± 2.6	9.6 ± 3.1	9.6 ± 3.2	9.1 ± 3.3	0.524	10.0 ± 2.3	10.0 ± 2.6	9.6 ± 2.9	9.0 ± 3.3	0.283
13 – apical anterior	4.5 ± 1.2	4.8 ± 1.5	4.2 ± 1.3	4.3 ± 1.7	0.193	7.7 ± 1.9	7.9 ± 2.0	6.1 ± 1.8^a^, ^b^	5.1 ± 2.1^a^, ^b^, ^c^	<0.001
14 – apical septal	4.8 ± 0.9	5.4 ± 1.3^a^	4.7 ± 1.3^b^	4.5 ± 1.4^b^	0.001	8.2 ± 1.8	8.6 ± 1.9	6.7 ± 1.6^a^, ^b^	5.3 ± 1.7^a^, ^b^, ^c^	<0.001
15 – apical inferior	6.7 ± 1.5	6.6 ± 2.1	6.2 ± 1.7	5.6 ± 1.8^a^	0.011	9.4 ± 2.1	9.6 ± 2.1	7.7 ± 2.2^a^, ^b^	6.2 ± 2.0^a^, ^b^, ^c^	<0.001
16 – apical lateral	7.0 ± 1.4	7.7 ± 2.1	7.2 ± 2.3	7.0 ± 2.7	0.419	8.1 ± 1.6	8.6 ± 1.9	7.8 ± 2.0	7.2 ± 2.5^b^	0.004
17 – apex	5.6 ± 1.3	6.2 ± 1.5	5.5 ± 1.5	5.1 ± 1.6^b^	0.001					

*Note*: *p*‐Values were derived from ANOVA and post‐hoc comparisons were carried out using the Holm‐Bonferroni method.

^a^ Significantly different from controls.

^b^ Significantly different from HFpEF.

^c^ Significantly different from HFmrEF.

**TABLE 3:** Long axis longitudinal displacement (mean ± standard deviation) for each LV segment.

	Epicardium					Endocardium				
Segment	Control	HFpEF (>55%)	HFmrEF (40−55%)	HFrEF (<40%)	*p*‐Value	Control	HFpEF (>55%)	HFmrEF (40−55%)	HFrEF (<40%)	*p*‐Value
1 – basal anterior	6.4 ± 2.0	4.2 ± 1.7^a^	4.6 ± 2.0^a^	4.8 ± 2.1^a^	<0.001	5.2 ± 1.7	4.0 ± 1.3^a^	4.3 ± 1.8^a^	4.3 ± 2.1	0.005
2 – basal anteroseptal	7.4 ± 2.3	5.9 ± 2.0^a^	5.6 ± 1.8^a^	5.3 ± 1.9^a^	<0.001	4.9 ± 1.8	3.8 ± 1.5^a^	4.0 ± 1.5^a^	4.3 ± 1.4	0.007
3 – basal inferoseptal	7.1 ± 2.3	5.7 ± 2.3^a^	5.0 ± 1.9^a^	4.8 ± 2.2^a^	<0.001	6.7 ± 2.1	5.0 ± 2.1^a^	5.0 ± 2.1^a^	4.8 ± 2.2^a^	<0.001
4 – basal inferior	9.8 ± 2.9	7.6 ± 2.8^a^	7.7 ± 2.8^a^	6.5 ± 2.5^a^	<0.001	8.5 ± 2.7	6.8 ± 2.8^a^	7.1 ± 2.8	6.4 ± 2.5^a^	<0.001
5 – basal inferolateral	9.9 ± 2.2	8.3 ± 2.6^a^	8.2 ± 2.9^a^	7.3 ± 2.4^a^	<0.001	9.1 ± 2.5	7.2 ± 2.1^a^	7.1 ± 2.8^a^	6.8 ± 2.5^a^	<0.001
6 – basal anterolateral	9.7 ± 2.8	7.5 ± 2.8^a^	7.9 ± 2.9^a^	7.6 ± 2.7^a^	<0.001	8.4 ± 2.7	6.1 ± 2.2^a^	7.1 ± 2.9	6.9 ± 2.7^a^	<0.001
7 – mid anterior	5.6 ± 1.8	4.3 ± 1.8^a^	4.6 ± 2.0^a^	5.2 ± 2.4	0.005	3.7 ± 1.4	3.8 ± 1.5	3.7 ± 1.9	4.5 ± 2.5	0.084
8 – mid anteroseptal	4.5 ± 1.6	3.8 ± 1.3	3.7 ± 1.5	3.7 ± 1.4	0.019	4.0 ± 1.1	4.3 ± 1.4	3.3 ± 1.3^a^, ^b^	3.0 ± 0.9^a^, ^b^	<0.001
9 – mid inferoseptal	4.4 ± 1.7	3.7 ± 1.7	3.6 ± 1.6	3.6 ± 1.6	0.029	3.5 ± 1.0	3.7 ± 1.3	3.0 ± 1.1	3.2 ± 1.4	0.033
10 – mid inferior	8.4 ± 2.7	6.8 ± 3.1^a^	7.3 ± 2.9	6.1 ± 2.4^a^	<0.001	6.3 ± 2.3	5.4 ± 2.2	5.8 ± 2.7	5.6 ± 2.5	0.302
11 – mid inferolateral	8.6 ± 2.1	8.6 ± 2.9	8.2 ± 3.1	7.7 ± 3.0	0.346	7.4 ± 2.5	7.0 ± 2.1	6.8 ± 3.0	7.3 ± 3.2	0.665
12 – mid anterolateral	9.0 ± 2.7	8.1 ± 3.4	8.4 ± 3.4	8.3 ± 3.3	0.542	6.8 ± 2.5	6.2 ± 2.2	6.9 ± 3.1	7.3 ± 3.3	0.265
13 – apical anterior	3.9 ± 1.4	4.2 ± 1.5	3.7 ± 1.2	3.9 ± 1.6	0.530	4.2 ± 1.5	4.6 ± 1.6	3.8 ± 1.5^b^	3.9 ± 1.9	0.058
14 – apical septal	3.6 ± 0.9	4.2 ± 1.2^a^	3.7 ± 1.2	3.4 ± 0.9^b^	0.002	4.9 ± 1.5	5.6 ± 1.7	4.5 ± 1.5^b^	3.6 ± 1.3^a^, ^b^, ^c^	<0.001
15 – apical inferior	4.9 ± 1.7	4.3 ± 2.0	4.7 ± 2.0	4.3 ± 1.8	0.318	4.2 ± 1.3	4.9 ± 1.4	4.0 ± 1.4^b^	4.0 ± 1.9^b^	0.008
16 – apical lateral	5.7 ± 1.4	6.2 ± 2.1	6.2 ± 2.4	6.4 ± 2.7	0.517	5.1 ± 1.3	5.5 ± 1.4	5.5 ± 2.0	6.0 ± 2.7	0.117
17 – apex	4.5 ± 1.0	5.1 ± 1.3	4.6 ± 1.4	4.5 ± 1.5	0.100					

*Note*: *p*‐values were derived from ANOVA and post‐hoc comparisons were carried out using the Holm‐Bonferroni method.

^a^ Significantly different from controls.

^b^ Significantly different from HFpEF.

^c^ Significantly different from HFmrEF.

**TABLE 4:** Long axis radial displacement (mean ± standard deviation) for each LV segment.

	Epicardium					Endocardium				
Segment	Control	HFpEF (>55%)	HFmrEF (40−55%)	HFrEF (<40%)	*p*‐Value	Control	HFpEF (>55%)	HFmrEF (40−55%)	HFrEF (<40%)	*p*‐Value
1 – basal anterior	9.2 ± 2.0	7.3 ± 2.6^a^	7.3 ± 2.3^a^	6.6 ± 2.1^a^	<0.001	10.8 ± 2.9	8.5 ± 3.0^a^	8.1 ± 3.0^a^	7.4 ± 2.7^a^	<0.001
2 – basal anteroseptal	3.9 ± 1.2	3.5 ± 1.2	3.3 ± 1.5	3.5 ± 1.6	0.118	5.5 ± 1.5	4.9 ± 1.3	4.6 ± 1.3^a^	4.3 ± 1.6^a^	<0.001
3 – basal inferoseptal	5.2 ± 1.3	4.0 ± 1.4^a^	3.9 ± 1.6^a^	3.7 ± 1.4^a^	<0.001	5.3 ± 1.8	4.7 ± 1.4	4.0 ± 1.4^a^	3.9 ± 1.8^a^	<0.001
4 – basal inferior	7.5 ± 1.9	6.6 ± 2.1^a^	6.3 ± 2.0^a^	4.9 ± 1.5^a^, ^b^, ^c^	<0.001	9.1 ± 2.1	8.0 ± 2.4^a^	7.2 ± 2.2^a^	5.8 ± 1.8^a^, ^b^, ^c^	<0.001
5 – basal inferolateral	7.7 ± 1.5	8.7 ± 2.4	7.5 ± 2.1^b^	6.6 ± 1.9^a^, ^b^	<0.001	9.8 ± 1.9	10.4 ± 2.7	8.6 ± 2.9^b^	7.5 ± 2.4^a^, ^b^	<0.001
6 – basal anterolateral	9.0 ± 2.0	9.4 ± 2.3	8.4 ± 2.3	7.3 ± 2.1^a^, ^b^	<0.001	11.3 ± 2.4	11.4 ± 2.9	10.0 ± 2.6^a^, ^b^	8.4 ± 2.7^a^, ^b^, ^c^	<0.001
7 – mid anterior	4.4 ± 1.2	4.0 ± 1.6	3.5 ± 1.4^a^	3.1 ± 1.2^a^, ^b^	<0.001	9.0 ± 2.4	8.2 ± 2.6	6.5 ± 2.4^a^, ^b^	4.8 ± 2.0^a^, ^b^, ^c^	<0.001
8 – mid anteroseptal	3.8 ± 1.2	3.6 ± 1.1	3.2 ± 1.4	3.5 ± 1.5	0.127	7.2 ± 1.9	6.5 ± 2.1	5.3 ± 2.0^a^, ^b^	4.5 ± 2.1^a^, ^b^	<0.001
9 – mid inferoseptal	4.0 ± 1.2	3.9 ± 1.4	3.4 ± 1.3^a^	3.4 ± 1.5	0.024	7.1 ± 1.6	6.4 ± 2.0	5.2 ± 1.6^a^, ^b^	4.4 ± 1.8^a^, ^b^	<0.001
10 – mid inferior	4.4 ± 1.2	4.5 ± 1.6	3.9 ± 1.5	3.4 ± 1.6^a^, ^b^	0.001	8.1 ± 1.6	7.8 ± 2.2	6.1 ± 1.5^a^, ^b^	4.6 ± 1.8^a^, ^b^, ^c^	<0.001
11 – mid inferolateral	4.6 ± 1.2	5.3 ± 1.7	4.6 ± 1.7	4.1 ± 1.6^b^	0.001	7.1 ± 1.7	8.3 ± 2.3^a^	6.5 ± 2.1^b^	5.2 ± 2.1^a^, ^b^, ^c^	<0.001
12 – mid anterolateral	4.6 ± 1.6	5.5 ± 1.5^a^	4.7 ± 1.5^b^	4.2 ± 1.8^b^	<0.001	7.6 ± 2.0	8.3 ± 2.2	6.8 ± 1.8^b^	5.6 ± 2.1^a^, ^b^, ^c^	<0.001
13 – apical anterior	2.8 ± 0.9	2.9 ± 1.1	2.5 ± 1.2	2.2 ± 1.1^a^, ^b^	0.007	6.8 ± 2.1	6.9 ± 2.1	5.2 ± 1.8^a^, ^b^	3.7 ± 1.8^a^, ^b^, ^c^	<0.001
14 – apical septal	3.6 ± 1.0	4.0 ± 1.2	3.3 ± 1.2^b^	3.2 ± 1.5	0.013	7.1 ± 1.7	7.0 ± 2.0	5.4 ± 1.8^a^, ^b^	4.3 ± 1.8^a^, ^b^, ^c^	<0.001
15 – apical inferior	5.0 ± 1.3	5.2 ± 1.9	4.5 ± 1.6	4.1 ± 1.9^b^	0.005	8.8 ± 2.1	8.7 ± 2.1	6.9 ± 2.3^a^, ^b^	5.2 ± 2.1^a^, ^b^, ^c^	<0.001
16 – apical lateral	4.4 ± 1.4	5.0 ± 1.5	4.2 ± 1.7^b^	3.4 ± 1.4^a^, ^b^, ^c^	<0.001	7.0 ± 1.7	7.5 ± 1.8	6.1 ± 1.8^a^, ^b^	4.6 ± 1.6^a^, ^b^, ^c^	<0.001
17 – apex	3.8 ± 1.2	4.1 ± 1.3	3.4 ± 1.2^b^	2.8 ± 1.1^a^, ^b^	<0.001					

*Note*: *p*‐Values were derived from ANOVA and post‐hoc comparisons were carried out using the Holm‐Bonferroni method.

^a^ Significantly different from controls.

^b^ Significantly different from HFpEF.

^c^ Significantly different from HFmrEF.

Short‐axis epicardial and endocardial displacement data (overall/circumferential/radial) are shown for the first 12 segments and for each patient group in Tables [Link], [Link], and S3. Short‐axis data for each patient was limited to basal and/or mid‐cavity slices. The greatest motion is seen in the endocardium, particularly in the normal direction (radial displacement). Reduced LV contraction in the HF groups is most obvious in the normal direction—notably, endocardial normal displacement for the HFrEF group is significantly smaller in all basal and mid‐cavity segments compared to the control group.

Boxplots were created to show the spread of mean displacement data for each patient group and are included in the Supplementary Material. Figure S1 shows boxplots for mean epicardial (Figure S1a) and endocardial (Figure S1b) displacement in the basal, mid‐cavity, apical, and apical cap segment levels determined from long‐axis data. Epicardial displacement was significantly smaller in all HF groups compared to the control group for the basal level (*p* < 0.001) and mid‐cavity level (*p* = 0.036 HFpEF, *p* = 0.017 HFmrEF, *p* < 0.001 HFrEF). There was no significant difference between HF groups and the control group for apical and apical cap epicardial displacement. Endocardial displacement was significantly smaller for all HF groups compared to the control group (*p* ≤ 0.001), except for HFpEF mid‐cavity and apical segment levels.

Boxplots for short‐axis basal and mid‐cavity segment displacements are shown in Figure S1c and S1d. No significant difference in epicardial displacement was observed between the HF groups and the control group. HF group endocardial displacements were all significantly smaller than the control group displacements for both basal (*p* = 0.026 HFpEF, *p* < 0.001 HFmrEF and HFrEF) and mid‐cavity levels (*p* = 0.002 HFpEF, *p* < 0.001 HFmrEF and HFrEF).

Male and female displacement data were also compared for each segment level and patient group. No significant difference was seen for all comparisons except for HFmrEF long‐axis apical epicardium (*p* = 0.046).

Motion trace analysis (Figure 3) confirmed that epicardial and endocardial motion beginning from the reference end‐diastolic phase is primarily towards the central axis and apex of the LV. This predominantly inward and apical motion of the LV is well known.^17^, ^35^, ^36^ Other examples are shown in Figure S2.

## 4 DISCUSSION

This analysis provides reference data for segment‐specific cardiac motion across a wide population, including those with and without HF and a range of LV function. Such data forms a reference point for the development of LV segment‐specific margin guidelines for cardiac radioablation. This data could also be used as a starting point for future cardiac‐respiratory motion phantom studies, which will be important for investigating the impact of motion on cardiac radioablation and for optimizing the treatment technique. Additionally, the cardiac‐specific motion data presented in this work could be applied during treatments where respiratory motion is separately accounted for with gating or tracking methods. Displacement data in this study is presented in a LV‐centric frame (longitudinal, circumferential, and radial displacements in the long‐ and short‐axis planes), eliminating the factor of variation in patient heart orientation. The data can be easily translated to the patient coordinate system used in radiotherapy (superior‐inferior [SI]/left‐right [LR]/anterior‐posterior [AP]) using the orientation of the LV long axis in 3D space. ITV margins normal to the epicardial and endocardial surface could also be generated from margining tools on most treatment planning systems.

Since there was no significant difference between the height, weight, or BSA between the groups, it is unlikely that the motion differences observed were due to these factors rather than the pathology itself. As an additional check, long‐axis overall displacement normalized by BSA was calculated (Table S4) and the observed significant differences were nearly identical to those seen in the raw data shown in Table 2.

Interesting comparisons can be made within each group between the different displacements in specific LV segments. For example, Table 2 shows that displacements in segment 2 (basal anteroseptal) and segment 3 (basal inferoseptal) are much smaller than in the rest of the basal segments, likely due to the LV outflow tract (see, Figure 2c,d). Planning margins may be set based on target location with larger margins required for targets in the basal segments compared to targets at the apex. The data in this work can provide upper bounds to the expected motion displacements. As expected, there is less motion in those with reduced function (HF patients with lower LVEF). Statistically significant motion reduction (compared to the control group) ranges from about 1 to 4 mm depending on the segment, with the greatest reduction seen in the HFrEF group. Cardiac radioablation is often in close proximity, if not directly adjacent to OARs such as the esophagus, stomach, liver, chest wall, great vessels, and proximal bronchial tree. Therefore, even a millimeter change in ITV contouring could result in improved target coverage or the difference between a tolerable OAR dose versus toxicity. Endocardium motion is also generally greater than epicardium motion in the normal direction—greater than a 4 mm difference in some segments for the control group but less pronounced in the HF groups as LVEF decreases. The difference between epicardial and endocardial ITV margins may only be 0 to 2 mm for HFrEF patients.

This reference data is especially important for segments that are particularly close to OARs. The apical segments are near the chest wall; the inferior segments are close to the stomach, liver, and esophagus; and the anterior segments are close to the pulmonary tree. A figure of the planning contours for a sample patient is included in the Supplementary Material (Figure S3), showing the location of these OARs relative to the heart. Planning organ at risk volume (PRV) margins were added for the proximal bronchial tree, esophagus, and stomach due to their radiosensitivity (shown in the figure). Late complications due to cardiac radioablation have been reported, including one patient who developed a gastropericardial fistula^37^ and another who developed an esophageal fistula.^38^ Deep inspiration breath hold (DIBH) has been used to reduce gastrointestinal dose in the treatment of VT originating from LV inferior scar.^39^ In patients with arrhythmias, the stomach‐heart distance was measured to be 7.5 ± 4.2 mm during end‐systole and 6.1 ± 3.6 mm during end‐diastole, where the mid‐inferior LV was most frequently the closest segment.^40^ In other words, 68% of patients will have their stomach located within 2.5 to 9.7 mm of the heart at end‐diastole. As a specific example, mean epicardial radial displacement for the mid inferior segment in a HFrEF patient is 3.4 ± 1.6 mm or less than 5 mm in 84% of patients. Half of this value (2.5 mm) could be applied as the cardiac portion of the ITV. Prusator et al. reported 6.7 mm (average + 1 standard deviation) of respiratory motion in the superior inferior direction for abdominal compression free breathing patients. Adding half this value (3.4 mm) to the cardiac component would yield a combined cardiac respiratory ITV margin of 5.9 mm. A PTV margin of between 3−5 mm to account for set up uncertainty would also be added, leading to a total margin of between 8.9 and 10.9 mm. In this case, target OAR overlap would exist for approximately 84% of patients. Compromises to target coverage would likely need to be made depending on the situation. Ideally, patient‐specific values from a CMR or cardiac 4DCT study would be used for planning—when not available, the mean values in these tables provide a reasonable estimate.

A previous study measured heart substructure motion using cardiac gated computed tomography, with the purpose of providing margin guidelines for cardiac radioablation.^41^ However, only average centroid displacement (0.51 ± 0.53 mm SI, 0.53 ± 0.43 mm LR, 1.20 ± 0.83 mm AP) and the ratio of maximum to minimum volume (range 1.15 to 1.54) were reported for the LV. Prusator et al. estimated the motion for cardiac radioablation targets located in various LV segments by measuring target trajectories on respiratory and cardiac 4DCT using rigid registration of each 4D phase to the reference (0%) phase.^42^ Mean (range) target motion across 11 patients from the cardiac 4DCT was 3.4 (1.0−4.8) mm LR, 4.3 (2.6−6.5) mm AP, and 4.1 (1.4−8.0) mm SI. The more specific regional LV myocardial displacements presented in the current study provide additional detailed motion data that should be considered for cardiac treatment planning margins at multiple cardiac locations. The described technique of tracking contour points across the cardiac cycle provides a good estimate of cardiac motion in different areas of the LV. However, this method only characterizes displacement in the short‐ or long‐axis planes and does not account for through‐plane motion. The twisting motion of the LV, for example, results in through‐plane motion in the long‐axis slices.^43^ During contraction, the LV apex and base are rotated counter‐clockwise and clockwise, respectively, as viewed from the apical end. Peak apical rotation in healthy humans is estimated to be −11.6 ± 3.8°, measured by MRI tagging, or −10.9 ± 3.3°, measured by speckle tracking echocardiography (STE), while peak basal rotation is 4.8 ± 1.7° (MRI tagging) or 4.6 ± 1.3° (STE).^44^ This corresponds to an estimate of rotational displacement on the order of 2 to 4 mm. Rotation at the equatorial level is minimal. Furthermore, rotation is reduced in patients with chronic HF and reduced ejection fraction.^45^

A limitation of this study is the reliance of contour accuracy on feature tracking displacement fields, which are generated from non‐rigid registration of cine images. Although manual tracings were completed on the reference end‐diastolic frame by an experienced interpreter, the registration process used to propagate the contours to all cardiac cycle images introduces additional uncertainty. Another limitation is the lack of data in regards to the presence and location of scar for the study subjects—a future study could investigate the relation between myocardial scar size/location and LV motion. This study also used limited short‐axis data (two slices per patient). Similar analysis using a full stack of short‐axis slices covering the heart from base to apex would provide valuable additional data.

Special consideration should be taken for patients with implanted ICDs, as image artifacts near the ICD will affect the ability to assess cardiac motion. Susceptibility artifacts can be caused by distortion of the MRI magnetic field surrounding the ICD generator and leads.^46^ BSSFP images (which are used in this study) also suffer from off resonance effects due to ICD‐induced magnetic field perturbations. For patients with left‐sided ICD systems, the anterior and apical segments of the LV are particularly affected by artifacts due to the proximity of the ICD generator.^47^ Motion evaluation employing MRI may be difficult for treatment targets located in these regions and population or disease‐specific norms may prove useful.

This work also focuses only on cardiac motion. Patient‐specific respiratory motion estimation has been well studied within the field of radiation therapy.^48^, ^49^, ^50^ Furthermore, technologies (such as respiratory‐correlated 4DCT) and experience at measuring respiratory motion exists within many radiation therapy departments worldwide. Heart motion and deformation due to respiration has been quantified by imaging at multiple positions in the breathing cycle, then using a rigid registration followed by non‐rigid registration algorithm.^51^ LV displacement due to respiratory motion should be accounted for separately and the effect of breathing on cardiac contraction motion should also be considered.^52^, ^53^, ^54^, ^55^, ^56^ Combined cardiac and respiratory motion could be evaluated in a future study using free‐breathing CMR. Alternative techniques for motion assessment could be explored in future studies, such as displacement encoding with stimulated echoes (DENSE), which encodes tissue displacement directly into the CMR signal.^57^, ^58^ Patient‐specific applications could also be considered, for example where treatment planning is based on the 17‐segment cardiac motion data for an individual patient.

**FIGURE 4:**
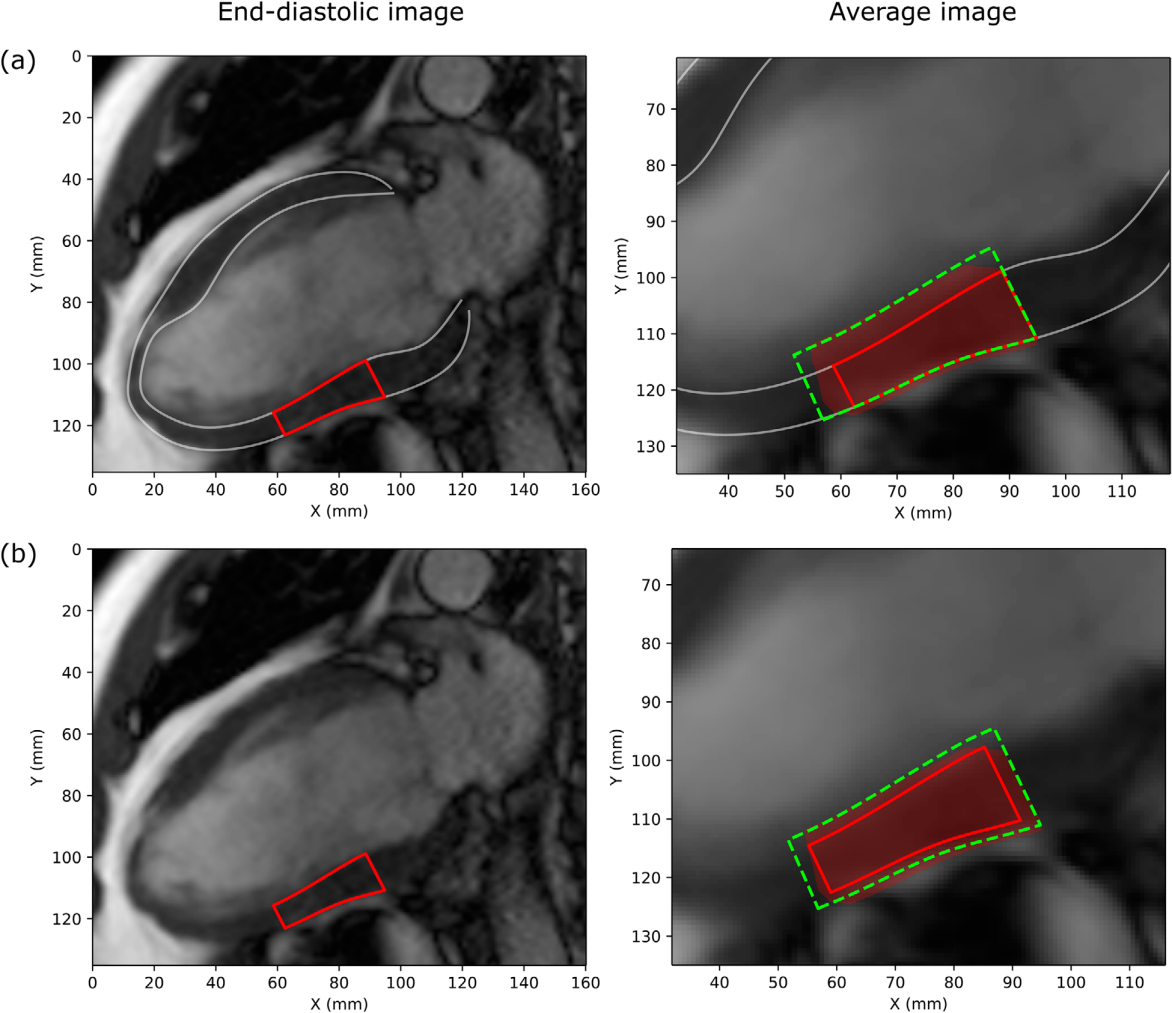
The applied ITV margin will depend on the imaging method. In figure (a), the LV and target are defined on the end‐diastolic image and the proposed ITV is shown for the case where the entire LV is matched on the average image (e.g., CBCT). Figure (b) shows an example where the target itself is used for matching and the ITV is composed of halved epicardial and endocardial radial/longitudinal displacements in all directions. The average image in this figure was generated by averaging all CMR phase images to approximate a CBCT (and the associated image blur) typically used during treatment. CBCT, cone beam computed tomography; CMR, cardiovascular magnetic resonance; dashed green outline, proposed ITV based on mean HFrEF displacement data; ITV, internal target volume; LV, left ventricle; red outline, target; red shaded area, actual area covered by target over cardiac cycle.

How the motion data presented in this work is used for margin guidance is a complicated problem that depends on several factors. Some of these factors include: the target size, the primary scans used for target delineation (cardiac/respiratory cine, gated or average), the degree of respiratory motion and the motion mitigation strategies employed (gating, tracking, abdominal compression), the treatment machine imaging systems and protocols used for treatment target matching, and the match priorities (i.e., is the whole ventricle matched or just the target segments). Examples of different applications of the applied data for ITV margin estimation are demonstrated (for cardiac motion only) in Figure 4, where in (a) an end‐diastolic image is matched to an average (with blur) cone beam computed tomography (CBCT) image, magnetic resonance (MR) image, etc. (using the entire LV as a match target) versus (b) where a target defined on an end‐diastolic image is matched to the center of an average CBCT/MR blurred image. When matching the entire LV (Figure 4a), the epicardial border would be aligned with the outer edge of the blurred LV in the average image—given knowledge of the primary direction of motion from the end‐diastolic phase (Figure 3), the ITV margins might be applied primarily in the inner and apical directions. When matching the target itself (Figure 4b), the target would likely be centered between the blurred epicardial and endocardial borders—in this case, the ITV margins applied isotropically using half the expected epicardial and endocardial displacements may be prudent. The examples shown in Figure 4 are segment 10 (mid inferior) for a HFrEF patient. The corresponding epicardial and endocardial mean displacements for the HFrEF group were used to create the proposed ITV. In Figure 4a where the entire LV is matched, the entire endocardial radial displacement of 4.6 mm is applied as the inner margin. There is no epicardial margin applied, since the epicardium is primarily moving inward. Similarly, the entire longitudinal displacements are applied for the apical margin (6.1 mm epicardium and 5.6 mm endocardium), while no basal margin is applied. In Figure 4b where the target is matched to the center of the blurred myocardium, the mean displacements are divided by two and applied in the corresponding radial and longitudinal directions: 1.7 mm for the epicardial margin, 2.3 mm for the endocardial margin, 3.05 mm (epicardial) and 2.8 (endocardial) for the apical and basal margins. The red shaded area in the figure shows the actual range of motion of the target over the cardiac cycle, which is well encompassed by the proposed ITV in these examples. In addition to this, a number of recently demonstrated technological developments in the areas of onboard imaging^59^, ^60^, ^61^ and treatment^60^, ^61^, ^62^, ^63^ will likely affect application of this data to the treatment process. For example, cardiac and/or respiratory gated imaging would allow for improved image quality and reduced motion blur—in these scenarios, the application of motion margins will depend on the chosen gating windows.

In general, respiratory motion is a relatively easier problem to deal with, as the higher amplitude and lower frequency motion makes motion compensation, such as gating or tracking, an easier task. Whether or not respiratory motion is specifically compensated for during treatment, patient‐specific respiratory motion is always incorporated into treatment planning since a respiratory 4DCT, at minimum, is usually performed for motion evaluation. Patient‐specific cardiac‐induced motion data is also preferable to have, but when not available, having some reference of region‐specific motions will be valuable.

The cardiac‐specific motion data would also be more valuable for treatments with separate respiratory motion compensation, and cardiac contraction is the sole contributor to internal motion. Assessment of cardiac‐induced motion in the presence of respiratory motion may require a separate analysis, since cardiac targets in reality are affected by complex interplay between the two sources of motion. Future planning studies will incorporate both respiratory and cardiac motion for a thorough investigation of the dosimetric effects of target motion and different treatment strategies.

## 5 CONCLUSIONS

Cardiac radioablation targeted at the LV should account for the motion of the area to be treated. Cardiac motion is variable depending on the specific region of the LV wall. The data presented in this study provides an estimate of epicardial and endocardial displacement for the 17 segments of the LV. This reference data can aid institutions performing cardiac radioablation in determining more specific cardiac motion margins.

Patient‐specific motion data is always preferable, especially since cardiac motion can have large inter‐patient variability. This study data mainly serves as a reference for cardiac‐induced motion in different heart regions, and in the absence of cardiac‐specific motion data—such as when only respiratory 4DCT is used for motion evaluation—may also help to guide margin decisions.

## AUTHOR CONTRIBUTIONS

Justin Poon analyzed the data and wrote the original manuscript. Imaging data was provided by Richard B. Thompson. The work was conducted under the supervision of Steven Thomas. All authors were involved with the interpretation of data and manuscript revision.

## CONFLICT OF INTEREST STATEMENT

H.C. has received an in‐kind software loan from SIEMENS Healthcare. D.S. has received honoraria for participation in AstraZeneca advisory boards and for presentations given at events sponsored by AstraZeneca, Bristol Myers Squibb, and Pfizer.

## Supporting information

Supporting Information

Supporting Information

Supporting Information

Supporting Information

Supporting Information

Supporting Information

Supporting Information

Supporting Information

Supporting Information

Supporting Information

## Data Availability

The data that support the findings of this study are owned by a third party and are not publicly available. Requests made to the corresponding author will be directed towards Richard Thompson.
