# Supplementary material for: Analysis of left ventricle regional myocardial motion for cardiac radioablation: Left ventricular motion analysis
Source: J Appl Clin Med Phys. 2024 Mar 17;25(5):e14333. doi: 10.1002/acm2.14333 (PMC11087184; doi:10.1002/acm2.14333)
Supplement: Supplementary file 1 — Supporting Information [file ACM2-25-e14333-s007.pdf]

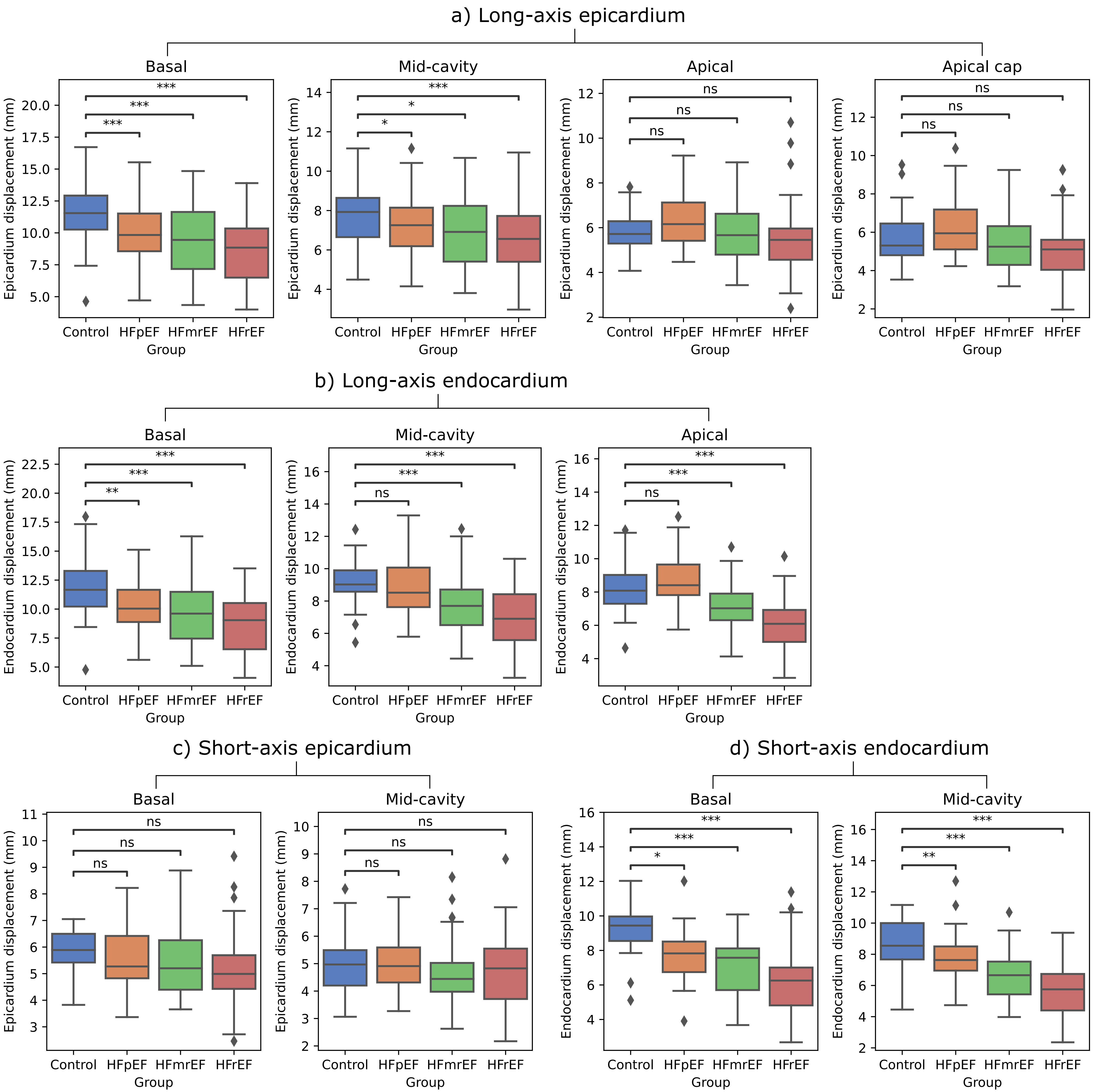

Supplementary Figure 1: Subject group mean segment level displacement boxplots for a) long-axis epicardium, b) long-axis endocardium, c) short-axis epicardium, and d) short-axis endocardium. Significance levels are indicated by ns: no significance, *: p<0.05, **: p<0.01, ***: p<0.001.
