# Supplementary figures and images for: Analysis of left ventricle regional myocardial motion for cardiac radioablation: Left ventricular motion analysis
Source: J Appl Clin Med Phys. 2024 Mar 17;25(5):e14333. doi: 10.1002/acm2.14333 (PMC11087184; doi:10.1002/acm2.14333)

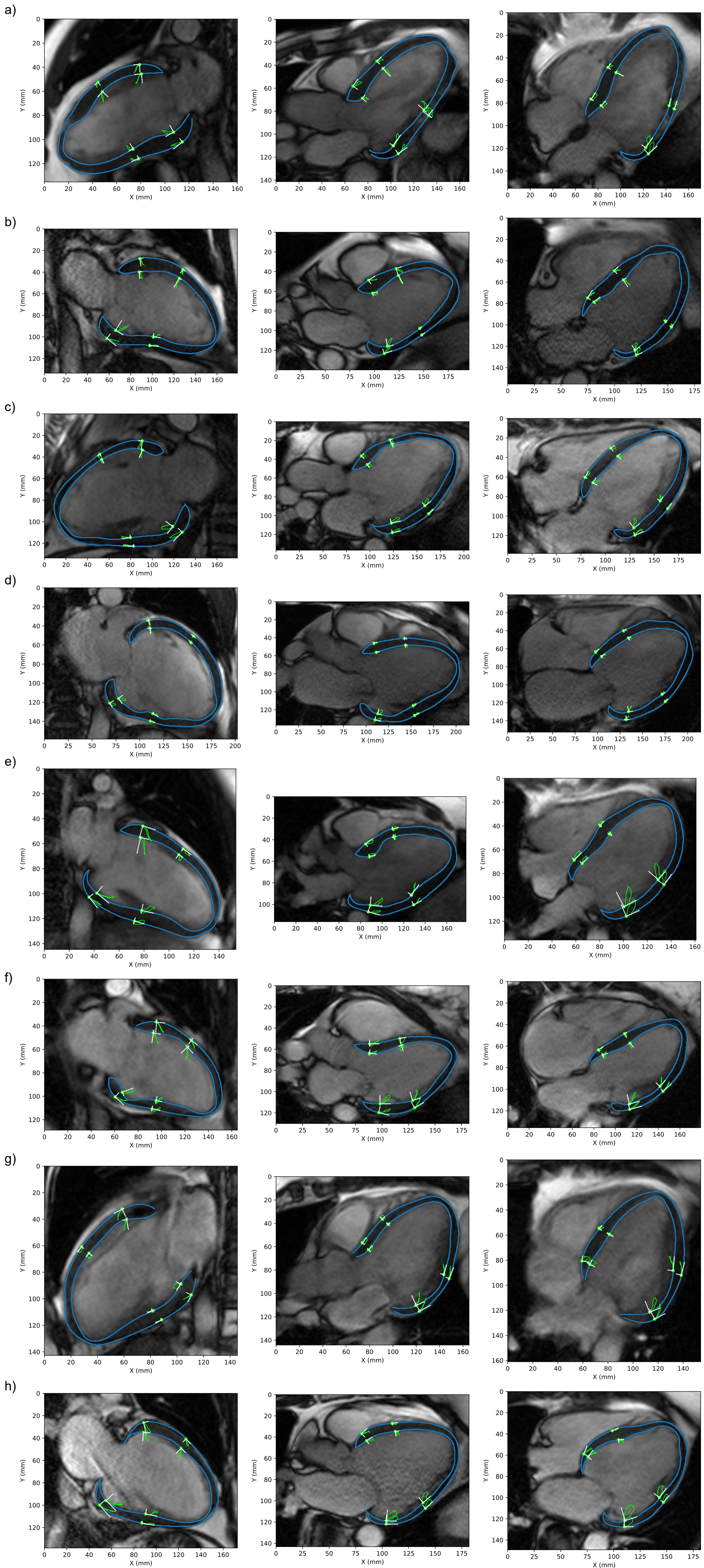

Supplement: Supplementary file 2 — Supporting Information [file ACM2-25-e14333-s009.pdf]
