# Supplementary material for: Analysis of left ventricle regional myocardial motion for cardiac radioablation: Left ventricular motion analysis
Source: J Appl Clin Med Phys. 2024 Mar 17;25(5):e14333. doi: 10.1002/acm2.14333 (PMC11087184; doi:10.1002/acm2.14333)
Supplement: Supplementary file 3 — Supporting Information [file ACM2-25-e14333-s006.pdf]

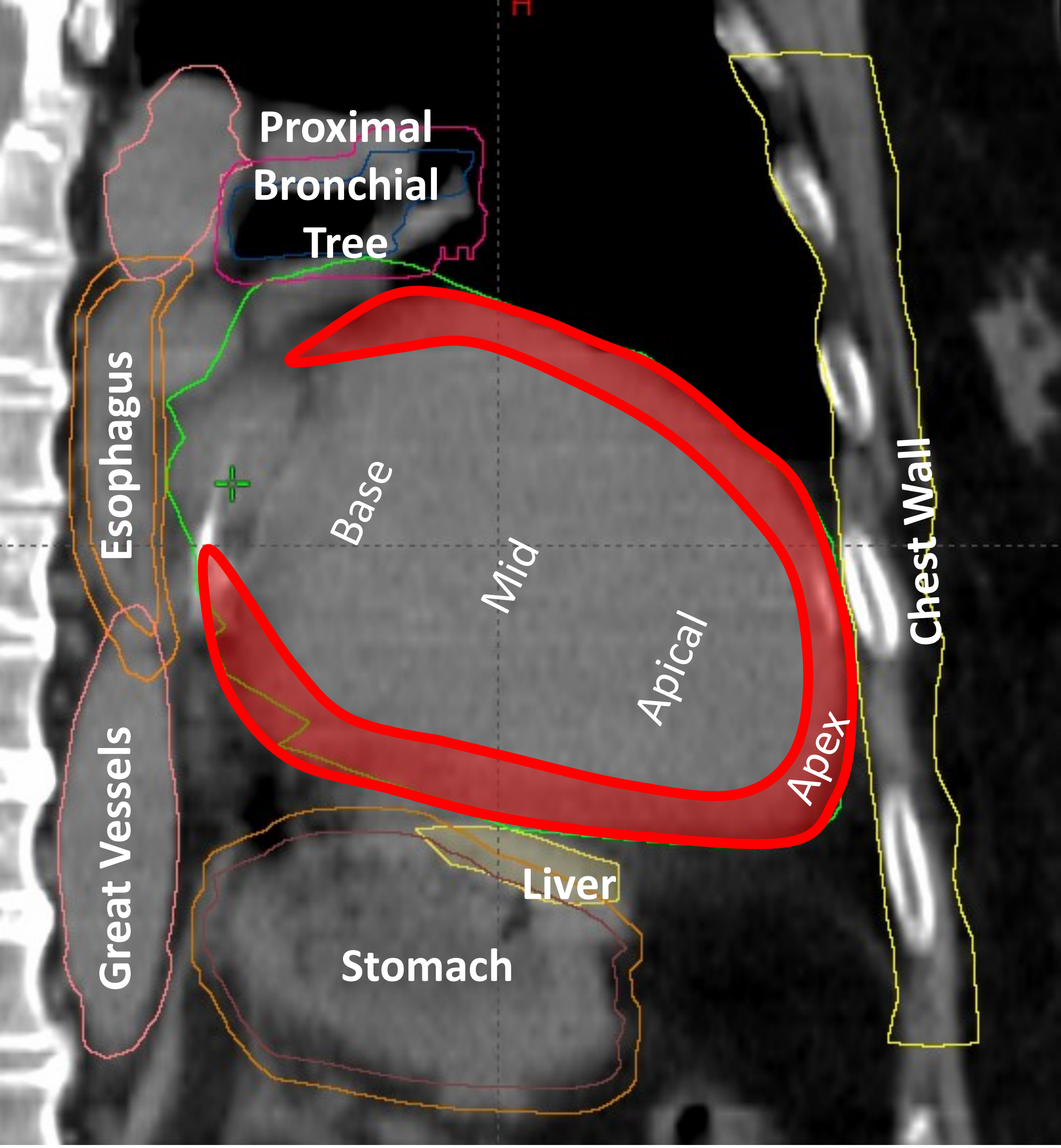

Supplementary Figure 3: Organs at risk (OARs) are shown for a sample cardiac radioablation patient. Planning organ at risk volume (PRV) margins were added for the proximal bronchial tree, esophagus, and stomach due to their radiosensitivity.
