# Supplementary material for: Analysis of left ventricle regional myocardial motion for cardiac radioablation: Left ventricular motion analysis
Source: J Appl Clin Med Phys. 2024 Mar 17;25(5):e14333. doi: 10.1002/acm2.14333 (PMC11087184; doi:10.1002/acm2.14333)
Supplement: Supplementary file 4 — Supporting Information [file ACM2-25-e14333-s003.rtf]

Supplementary Table 1 Short axis overall displacement (mean ± standard deviation) for each LV segment.
 	Epicardium	Endocardium	
Segment	Control	HFpEF
(>55%)	HFmrEF
(40–55%)	HFrEF
(<40%)	p-value	Control	HFpEF
(>55%)	HFmrEF
(40–55%)	HFrEF
(<40%)	p-value	
1 – basal anterior	6.8 ± 1.8	5.3 ± 1.8	5.2 ± 1.5*	5.4 ± 1.7*	0.004	11.5 ± 2.3	9.1 ± 3.1	7.6 ± 2.1*	7.4 ± 2.8*	<0.001	
2 – basal anteroseptal	4.4 ± 0.8	3.9 ± 1.3	3.9 ± 1.2	4.1 ± 1.1	0.301	8.5 ± 1.9	7.4 ± 2.0	4.8 ± 1.7*†	5.4 ± 1.7*†	<0.001	
3 – basal inferoseptal	4.8 ± 0.8	4.6 ± 1.2	4.8 ± 1.6	4.6 ± 1.5	0.895	6.5 ± 1.1	6.0 ± 1.3	5.7 ± 2.1	5.1 ± 1.6*	0.030	
4 – basal inferior	5.8 ± 1.2	6.0 ± 1.6	6.0 ± 1.8	5.6 ± 2.2	0.870	8.8 ± 2.0	7.4 ± 2.0	7.9 ± 2.8	6.4 ± 2.7*	0.007	
5 – basal inferolateral	6.4 ± 1.3	6.8 ± 1.7	6.4 ± 1.7	6.3 ± 2.6	0.876	9.1 ± 2.2	7.3 ± 2.7	8.7 ± 2.5	6.9 ± 3.0*	0.012	
6 – basal anterolateral	6.8 ± 1.3	6.6 ± 1.7	6.0 ± 1.4	5.9 ± 2.5	0.304	10.2 ± 2.1	8.9 ± 2.9	8.5 ± 2.0*	7.3 ± 3.1*	0.002	
7 – mid anterior	5.5 ± 1.4	4.9 ± 1.2	4.5 ± 1.1*	4.3 ± 1.5*	<0.001	9.8 ± 2.4	8.5 ± 2.5*	6.9 ± 2.0*†	5.7 ± 2.1*†‡	<0.001	
8 – mid anteroseptal	4.5 ± 1.0	3.9 ± 0.9*	3.7 ± 1.3*	3.9 ± 1.3	0.010	9.4 ± 2.2	8.0 ± 2.2*	6.5 ± 2.4*†	5.5 ± 2.4*†‡	<0.001	
9 – mid inferoseptal	4.3 ± 0.8	4.4 ± 1.4	4.5 ± 1.6	4.6 ± 1.7	0.754	8.0 ± 1.7	7.0 ± 1.6*	6.1 ± 2.0*†	5.5 ± 2.2*†	<0.001	
10 – mid inferior	4.8 ± 1.2	5.3 ± 1.7	5.4 ± 1.7	5.3 ± 1.9	0.329	8.2 ± 1.5	7.3 ± 1.7*	6.7 ± 2.1*	5.8 ± 2.2*†	<0.001	
11 – mid inferolateral	5.1 ± 1.5	5.8 ± 1.6	5.6 ± 1.6	5.5 ± 1.8	0.222	8.3 ± 1.8	7.5 ± 2.1	7.0 ± 2.1*	6.0 ± 2.1*†	<0.001	
12 – mid anterolateral	5.2 ± 1.5	5.5 ± 1.3	5.1 ± 1.2	4.9 ± 1.6	0.142	8.4 ± 2.2	7.9 ± 2.0	6.9 ± 1.7*†	5.8 ± 1.8*†‡	<0.001	
p-values were derived from ANOVA and post-hoc comparisons were carried out using the Holm-Bonferroni method.
*Significantly different from controls
†Significantly different from HFpEF
‡Significantly different from HFmrEF
