# Supplementary material for: Analysis of left ventricle regional myocardial motion for cardiac radioablation: Left ventricular motion analysis
Source: J Appl Clin Med Phys. 2024 Mar 17;25(5):e14333. doi: 10.1002/acm2.14333 (PMC11087184; doi:10.1002/acm2.14333)
Supplement: Supplementary file 5 — Supporting Information [file ACM2-25-e14333-s008.rtf]

Supplementary Table 2 Short axis circumferential displacement (mean ± standard deviation) for each LV segment.
 	Epicardium	Endocardium 	
Segment	Control	HFpEF
(>55%)	HFmrEF
(40–55%)	HFrEF
(<40%)	p-value	Control	HFpEF
(>55%)	HFmrEF
(40–55%)	HFrEF
(<40%)	p-value	
1 – basal anterior	3.1 ± 0.9	3.2 ± 1.5	3.0 ± 0.7	2.6 ± 1.1	0.209	3.8 ± 1.2	3.6 ± 1.7	3.0 ± 1.1	2.8 ± 1.0*	0.031	
2 – basal anteroseptal	3.0 ± 0.7	2.8 ± 0.9	2.8 ± 0.9	2.8 ± 1.0	0.764	5.2 ± 1.4	4.8 ± 2.3	3.0 ± 1.2*†	3.4 ± 1.4*	<0.001	
3 – basal inferoseptal	3.8 ± 1.0	4.1 ± 1.1	4.2 ± 1.6	3.9 ± 1.5	0.810	4.0 ± 0.8	3.7 ± 1.5	4.5 ± 2.2	3.8 ± 1.7	0.422	
4 – basal inferior	4.8 ± 1.0	4.8 ± 1.5	5.0 ± 1.8	5.2 ± 2.2	0.885	4.7 ± 1.4	3.9 ± 1.0	5.2 ± 2.3	5.0 ± 2.6	0.291	
5 – basal inferolateral	4.9 ± 1.1	4.5 ± 1.7	4.7 ± 1.6	4.9 ± 2.4	0.910	4.3 ± 1.5	3.8 ± 1.4	4.6 ± 1.7	4.5 ± 2.5	0.665	
6 – basal anterolateral	3.9 ± 1.2	3.1 ± 1.2	3.5 ± 1.0	3.4 ± 1.8	0.346	3.9 ± 1.6	2.8 ± 1.5	3.5 ± 1.5	3.2 ± 1.5	0.223	
7 – mid anterior	2.9 ± 1.1	3.0 ± 1.2	2.7 ± 0.9	2.7 ± 1.3	0.436	3.3 ± 0.9	3.9 ± 1.3*	3.0 ± 1.1†	2.8 ± 1.2*†	<0.001	
8 – mid anteroseptal	3.2 ± 1.0	2.9 ± 0.9	2.6 ± 0.9*	2.7 ± 1.0	0.015	5.3 ± 1.8	5.0 ± 2.1	3.9 ± 1.9*†	3.4 ± 1.7*†	<0.001	
9 – mid inferoseptal	3.3 ± 0.7	3.6 ± 1.3	3.6 ± 1.4	3.9 ± 1.6	0.129	4.6 ± 1.8	4.4 ± 1.6	4.1 ± 1.8	4.1 ± 1.9	0.564	
10 – mid inferior	3.8 ± 1.2	4.2 ± 1.7	4.3 ± 1.7	4.6 ± 1.9	0.116	4.7 ± 1.5	4.3 ± 1.3	4.4 ± 1.8	4.5 ± 2.2	0.728	
11 – mid inferolateral	3.6 ± 1.3	3.7 ± 1.2	4.0 ± 1.4	4.3 ± 1.9	0.107	4.0 ± 1.1	3.8 ± 1.4	3.9 ± 1.4	3.9 ± 1.7	0.909	
12 – mid anterolateral	2.8 ± 1.2	3.0 ± 1.2	3.0 ± 1.0	3.2 ± 1.5	0.546	3.2 ± 1.1	3.6 ± 1.3	3.0 ± 1.3	3.0 ± 1.3	0.059	
p-values were derived from ANOVA and post-hoc comparisons were carried out using the Holm-Bonferroni method.
*Significantly different from controls
†Significantly different from HFpEF
‡Significantly different from HFmrEF
