# Supplementary material for: Analysis of left ventricle regional myocardial motion for cardiac radioablation: Left ventricular motion analysis
Source: J Appl Clin Med Phys. 2024 Mar 17;25(5):e14333. doi: 10.1002/acm2.14333 (PMC11087184; doi:10.1002/acm2.14333)
Supplement: Supplementary file 6 — Supporting Information [file ACM2-25-e14333-s002.rtf]

Supplementary Table 3 Short axis radial displacement (mean ± standard deviation) for each LV segment.
 	Epicardium	Endocardium 	
Segment	Control	HFpEF
(>55%)	HFmrEF
(40–55%)	HFrEF
(<40%)	p-value	Control	HFpEF
(>55%)	HFmrEF
(40–55%)	HFrEF
(<40%)	p-value	
1 – basal anterior	6.5 ± 1.7	4.8 ± 1.7*	4.6 ± 1.8*	4.9 ± 1.7*	<0.001	11.0 ± 2.4	8.5 ± 3.0*	7.1 ± 2.2*	7.0 ± 2.8*	<0.001	
2 – basal anteroseptal	3.9 ± 0.8	3.3 ± 1.1	3.0 ± 1.2*	3.3 ± 1.0	0.032	7.2 ± 1.7	5.8 ± 1.3*	4.0 ± 1.7*†	4.5 ± 1.5*†	<0.001	
3 – basal inferoseptal	3.6 ± 0.9	3.0 ± 1.0	3.0 ± 1.2	2.9 ± 1.0	0.114	5.9 ± 1.2	5.3 ± 1.1	4.2 ± 1.4*	3.8 ± 1.5*†	<0.001	
4 – basal inferior	4.1 ± 1.1	4.2 ± 1.3	3.8 ± 1.3	2.9 ± 0.9*†‡	<0.001	8.3 ± 2.0	6.9 ± 1.9	6.5 ± 2.2*	4.8 ± 1.7*†‡	<0.001	
5 – basal inferolateral	4.5 ± 1.4	5.3 ± 1.6	4.8 ± 1.0	4.2 ± 1.5	0.087	8.5 ± 2.3	6.8 ± 2.5	7.6 ± 2.4	5.4 ± 2.2*‡	<0.001	
6 – basal anterolateral	5.7 ± 1.3	6.1 ± 1.7	5.3 ± 1.2	5.0 ± 2.2	0.170	9.7 ± 2.1	8.7 ± 2.8	8.0 ± 1.9*	6.8 ± 2.9*	<0.001	
7 – mid anterior	5.1 ± 1.4	4.2 ± 1.3*	3.9 ± 1.2*	3.6 ± 1.3*†	<0.001	9.4 ± 2.3	7.7 ± 2.5*	6.4 ± 2.0*†	5.1 ± 2.0*†‡	<0.001	
8 – mid anteroseptal	3.6 ± 0.9	3.2 ± 0.9	3.1 ± 1.2	3.2 ± 1.1	0.033	7.9 ± 1.7	6.3 ± 1.7*	5.3 ± 2.0*†	4.4 ± 2.0*†‡	<0.001	
9 – mid inferoseptal	3.6 ± 0.9	3.2 ± 1.1	3.1 ± 1.2	3.0 ± 1.0*	0.038	7.1 ± 1.4	6.0 ± 1.4*	5.1 ± 1.6*†	4.1 ± 1.7*†‡	<0.001	
10 – mid inferior	3.6 ± 1.0	3.9 ± 1.1	3.7 ± 1.4	2.9 ± 1.3*†‡	0.002	7.3 ± 1.8	6.4 ± 1.8	5.6 ± 1.9*	4.3 ± 1.5*†‡	<0.001	
11 – mid inferolateral	4.1 ± 1.3	4.8 ± 1.4*	4.3 ± 1.4	3.7 ± 1.2†	<0.001	7.7 ± 2.0	6.9 ± 2.0	6.4 ± 2.0*	5.0 ± 1.7*†‡	<0.001	
12 – mid anterolateral	4.7 ± 1.5	4.9 ± 1.4	4.5 ± 1.2	3.9 ± 1.3†	0.006	8.0 ± 2.4	7.2 ± 2.1	6.4 ± 1.8*	5.2 ± 1.8*†‡	<0.001	
p-values were derived from ANOVA and post-hoc comparisons were carried out using the Holm-Bonferroni method.
*Significantly different from controls
†Significantly different from HFpEF
‡Significantly different from HFmrEF
