# Supplementary material for: Analysis of left ventricle regional myocardial motion for cardiac radioablation: Left ventricular motion analysis
Source: J Appl Clin Med Phys. 2024 Mar 17;25(5):e14333. doi: 10.1002/acm2.14333 (PMC11087184; doi:10.1002/acm2.14333)
Supplement: Supplementary file 7 — Supporting Information [file ACM2-25-e14333-s010.rtf]

Supplementary Table 4 Long axis overall displacement (mean ± standard deviation) normalized by body surface area (BSA).
 	Epicardium	Endocardium 	
Segment	Control	HFpEF
(>55%)	HFmrEF
(40–55%)	HFrEF
(<40%)	p-value	Control	HFpEF
(>55%)	HFmrEF
(40–55%)	HFrEF
(<40%)	p-value	
1 – basal anterior	5.9 ± 1.4	4.2 ± 1.6*	4.4 ± 1.5*	4.1 ± 1.5*	<0.001	6.4 ± 1.7	4.7 ± 1.7*	4.7 ± 1.6*	4.3 ± 1.7*	<0.001	
2 – basal anteroseptal	4.1 ± 1.3	3.3 ± 1.1*	3.2 ± 1.0*	3.0 ± 1.1*	<0.001	3.6 ± 1.0	3.0 ± 0.9*	3.0 ± 0.8*	2.9 ± 0.9*	<0.001	
3 – basal inferoseptal	4.5 ± 1.3	3.4 ± 1.2*	3.2 ± 1.3*	2.9 ± 1.2*	<0.001	4.3 ± 1.2	3.3 ± 1.1*	3.2 ± 1.1*	3.0 ± 1.3*	<0.001	
4 – basal inferior	6.6 ± 1.9	5.1 ± 1.9*	5.3 ± 1.8*	4.2 ± 1.5*†‡	<0.001	6.4 ± 1.6	5.2 ± 1.8*	5.2 ± 1.7*	4.3 ± 1.5*†‡	<0.001	
5 – basal inferolateral	6.7 ± 1.5	6.1 ± 1.8	5.8 ± 1.8*	5.0 ± 1.5*†‡	<0.001	6.9 ± 1.5	6.2 ± 1.6	5.7 ± 1.9*	5.0 ± 1.6*†	<0.001	
6 – basal anterolateral	7.0 ± 1.7	6.0 ± 1.9*	6.0 ± 1.9*	5.2 ± 1.7*	<0.001	7.2 ± 1.6	6.4 ± 1.8*	6.3 ± 1.9*	5.3 ± 1.8*†‡	<0.001	
7 – mid anterior	3.6 ± 0.9	2.8 ± 1.1*	2.8 ± 1.1*	2.9 ± 1.1*	<0.001	5.0 ± 1.3	4.4 ± 1.5	3.7 ± 1.4*	3.2 ± 1.4*†	<0.001	
8 – mid anteroseptal	2.7 ± 0.8	2.5 ± 0.8	2.3 ± 0.9	2.4 ± 0.9	0.085	4.1 ± 1.0	3.8 ± 1.2	3.1 ± 1.0*†	2.6 ± 1.1*†	<0.001	
9 – mid inferoseptal	3.0 ± 0.9	2.6 ± 0.9	2.4 ± 1.0*	2.4 ± 1.0*	0.002	4.0 ± 0.8	3.6 ± 1.2	3.0 ± 0.9*†	2.6 ± 1.1*†	<0.001	
10 – mid inferior	4.9 ± 1.5	4.0 ± 1.5*	4.2 ± 1.4*	3.5 ± 1.2*‡	<0.001	5.3 ± 1.2	4.7 ± 1.4*	4.3 ± 1.4*	3.6 ± 1.3*†‡	<0.001	
11 – mid inferolateral	5.1 ± 1.2	5.0 ± 1.6	4.8 ± 1.5	4.3 ± 1.5*	0.037	5.3 ± 1.2	5.2 ± 1.4	4.8 ± 1.6	4.4 ± 1.7*	0.015	
12 – mid anterolateral	5.3 ± 1.4	4.9 ± 1.8	4.9 ± 1.7	4.5 ± 1.7	0.187	5.2 ± 1.3	5.1 ± 1.5	5.0 ± 1.6	4.5 ± 1.7	0.119	
13 – apical anterior	2.4 ± 0.8	2.4 ± 0.8	2.2 ± 0.7	2.1 ± 0.8	0.190	4.0 ± 1.1	4.0 ± 1.2	3.2 ± 1.1*†	2.6 ± 1.0*†‡	<0.001	
14 – apical septal	2.5 ± 0.6	2.7 ± 0.7	2.4 ± 0.6	2.2 ± 0.7†	0.004	4.3 ± 1.0	4.3 ± 1.1	3.5 ± 0.9*†	2.7 ± 0.9*†‡	<0.001	
15 – apical inferior	3.6 ± 1.0	3.3 ± 1.1	3.2 ± 1.0	2.8 ± 1.0*	0.004	4.9 ± 1.3	4.8 ± 1.1	4.0 ± 1.3*†	3.2 ± 1.1*†‡	<0.001	
16 – apical lateral	3.7 ± 0.9	3.9 ± 1.2	3.7 ± 1.1	3.5 ± 1.3	0.473	8.1 ± 1.6	8.6 ± 1.9	7.8 ± 2.0	7.2 ± 2.5†	0.004	
17 – apex	3.0 ± 0.9	3.1 ± 0.8	2.8 ± 0.7	2.5 ± 0.8†	0.002						
p-values were derived from ANOVA and post-hoc comparisons were carried out using the Holm-Bonferroni method.
*Significantly different from controls
†Significantly different from HFpEF
‡Significantly different from HFmrEF
