# Supplementary material for: Analysis of left ventricle regional myocardial motion for cardiac radioablation: Left ventricular motion analysis
Source: J Appl Clin Med Phys. 2024 Mar 17;25(5):e14333. doi: 10.1002/acm2.14333 (PMC11087184; doi:10.1002/acm2.14333)
Supplement: Supplementary file 8 — Supporting Information [file ACM2-25-e14333-s004.docx]

## Contour point segmentation

*Long Axis*

Contour points were automatically segmented based on the slice view and point locations. In the long-axis slice analysis workflow, the long axis of the LV was first defined in 3D space by identifying three points on the end-diastolic phase inner contour: the 2 basal endpoints and the apex of the endocardial border (Supplementary Figure a). The contour endpoints were identified as the anterior and inferior base points according to their left, posterior, superior (LPS) coordinates. The mid-point of the inferior and anterior base was taken as the mid-base, and the apex was automatically determined by finding the furthest point on the inner contour from the mid-base. The vector from the apex to the mid-base (in LPS coordinates) was defined as the LV long axis (Supplementary Figure a). The contour points were then divided into 7 segments: 3 segments on each side of the long axis, divided equally from the endocardial apex to the mid-base, and the apical cap segment from the endocardial to epicardial apex (Supplementary Figure b-d). The segments were numbered based on the specification of the slice as a 2-, 3-, or 4-chamber view – segments 1, 4, 7, 10, 13, 15, and 17 for a 2-chamber view; segments 2, 5, 8, 11, 14, 16, and 17 for a 3-chamber view; and segments 3, 6, 9, 12, 14, 16, and 17 for a 4-chamber view. The orientation of the contour was determined based on the identified anterior and inferior base points, allowing for assignment of each contour point to the proper segment. Contour point displacement data was stored for each segment, and analysis of the 2-, 3-, and 4-chamber views collectively provided enough data to cover all 17 segments.

*Short Axis*

When analyzing a short-axis slice, the slice level (basal, mid-cavity, or apical) must be determined so that the contour points may be divided into the proper segments. After defining the LV long axis (using a long-axis slice), the level of a short-axis slice could then be determined based on the location of the slice plane intersection with the long axis – basal, mid-cavity, or apical for the upper, middle, and lower thirds of the long axis, respectively. The second step was to select the point on the short-axis slice which marks the anterior insertion of the right ventricle (RV) wall into the LV (Supplementary Figure e-f). This point defines the border between the first (anterior) and second (antero-septal for a basal or mid-cavity slice and septal for an apical slice) segments, creating a starting point from which the contour points could be divided into the proper segments. Contour points were allocated into equal arc segments measured from the centroid of the endocardial border, with the number of segments and their labels based on the slice level: 6 segments (1 – 6) for a basal slice, 6 segments (7 – 12) for a mid-cavity slice, or 4 segments (13 – 16) for an apical slice (Supplementary Figure e-f).


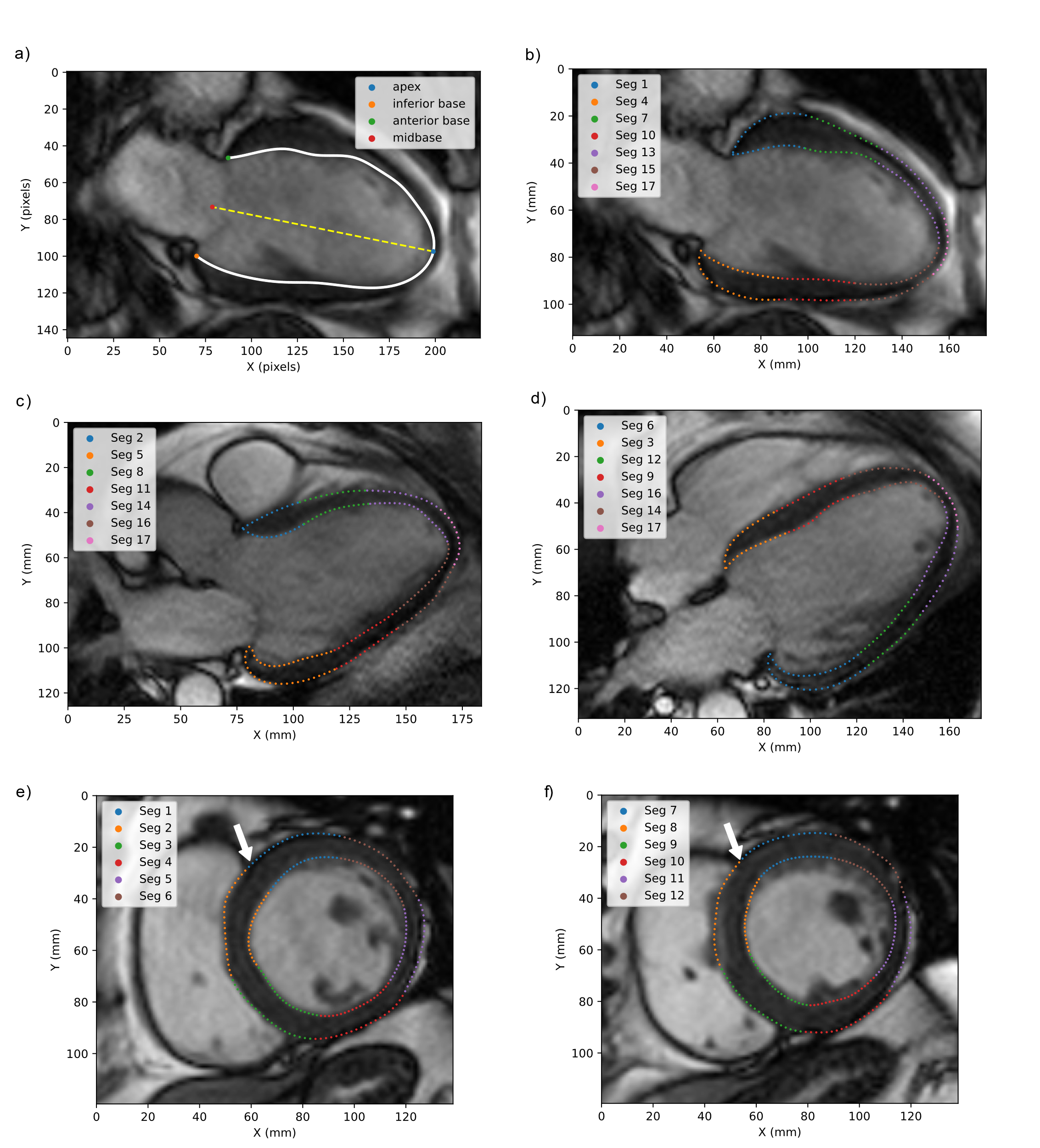


**Supplementary Figure** Contour point segmentation for a sample patient. Figure (a) shows an example of the automatically-defined LV long axis used for the segmentation. Figure (b, c, and d) show the resulting segmentation of the 2-chamber, 3-chamber, and 4-chamber view long-axis slices, respectively. Figure (e and f) show the segmentation for basal and mid-cavity short-axis slices, respectively. The arrows indicate the RV insertion point that marks the border between the first and second segments for each short-axis slice. Only a subset of the contour points is shown for visibility purposes.
